# Alterations in vasodilator-stimulated phosphoprotein (VASP) phosphorylation: associations with asthmatic phenotype, airway inflammation and β_2_-agonist use

**DOI:** 10.1186/1465-9921-7-25

**Published:** 2006-02-15

**Authors:** Annette T Hastie, Min Wu, Gayle C Foster, Gregory A Hawkins, Vikas Batra, Katherine A Rybinski, Rosemary Cirelli, James G Zangrilli, Stephen P Peters

**Affiliations:** 1 Department of Internal Medicine, & Center for Human Genomics, Wake Forest University School of Medicine, Winston-Salem, NC, USA; 2 Department of Medicine, Thomas Jefferson University, Philadelphia, PA, USA

## Abstract

**Background:**

Vasodilator-stimulated phosphoprotein (VASP) mediates focal adhesion, actin filament binding and polymerization in a variety of cells, thereby inhibiting cell movement. Phosphorylation of VASP via cAMP and cGMP dependent protein kinases releases this "brake" on cell motility. Thus, phosphorylation of VASP may be necessary for epithelial cell repair of damage from allergen-induced inflammation. Two hypotheses were examined: (1) injury from segmental allergen challenge increases VASP phosphorylation in airway epithelium in asthmatic but not nonasthmatic normal subjects, (2) regular *in vivo *β_2_-agonist use increases VASP phosphorylation in asthmatic epithelium, altering cell adhesion.

**Methods:**

Bronchial epithelium was obtained from asthmatic and non-asthmatic normal subjects before and after segmental allergen challenge, and after regularly inhaled albuterol, in three separate protocols. VASP phosphorylation was examined in Western blots of epithelial samples. DNA was obtained for β_2_-adrenergic receptor haplotype determination.

**Results:**

Although VASP phosphorylation increased, it was not significantly greater after allergen challenge in asthmatics or normals. However, VASP phosphorylation in epithelium of nonasthmatic normal subjects was double that observed in asthmatic subjects, both at baseline and after challenge. Regularly inhaled albuterol significantly increased VASP phosphorylation in asthmatic subjects in both unchallenged and antigen challenged lung segment epithelium. There was also a significant increase in epithelial cells in the bronchoalveolar lavage of the unchallenged lung segment after regular inhalation of albuterol but not of placebo. The haplotypes of the β_2_-adrenergic receptor did not appear to associate with increased or decreased phosphorylation of VASP.

**Conclusion:**

Decreased VASP phosphorylation was observed in epithelial cells of asthmatics compared to nonasthmatic normals, despite response to β-agonist. The decreased phosphorylation does not appear to be associated with a particular β_2_-adrenergic receptor haplotype. The observed decrease in VASP phosphorylation suggests greater inhibition of actin reorganization which is necessary for altering attachment and migration required during epithelial repair.

## Background

Attachment and migration of airway epithelial cells is an important aspect of repair of injury induced by allergens and other agents in asthma. Cytoskeletal reorganization, mediated through actin filament remodeling, is required to facilitate attachment and migration of epithelial cells into damaged areas. The vasodilator-stimulated phosphoprotein (VASP) binds to profilin, F- and G-actin, and mediates actin assembly, bundling and attachment at focal adhesions [1,2]. VASP and two other members of the ENA/VASP family in mammalian cells, MENA (mammalian ENAbled) and EVL (ENA/VASP-like) slow or inhibit fibroblast motility by altering the dynamics of actin filament structure [3,4]. However, MENA and EVL, despite shared molecular similarities and normal expression, did not substitute for VASP and reverse compromised cell motility in a wound healing assay of VASP-/- fibroblasts [5].

VASP phosphorylation occurs on two serine and one threonine (Ser 157, Ser 239 and Thr 278) through cAMP and cGMP dependent protein kinases A and G [6-9], and as recently observed in smooth muscle cells, protein kinase C [10]. Phosphorylation by cAMP protein kinase preferentially occurs at Ser 157, whereas phosphorylation by cGMP protein kinase preferentially occurs at Ser 239, both kinases secondarily acting on Thr 278 [7]. The Ser 157 phosphorylation results in decreased migration of the VASP molecule from 46 KD to 50 KD in SDS-gel electrophoresis [6-9]. Functionally, phosphorylation of VASP regulates its interaction with actin, and correlates with detachment and spreading of fibroblasts and epithelial cells [3,4,9,11,12]. Thus, the ratio of the 50 KD to the 46 KD molecular form of VASP within a cell sample provides a measure of VASP phosphorylation and indicates active remodeling of the actin cytoskeleton during cell attachment and migration.

We postulated that repair of injury to the airway epithelium during antigen-induced inflammation would involve enhanced phosphorylation of VASP in order to mediate actin cytoskeleton remodeling and cell migration into damaged areas. We further hypothesized that inhalation of the β-agonist albuterol, would also increase phosphorylation of VASP by stimulation of cAMP-dependent protein kinase activity, and result in altered epithelial cell attachment and mobility. These hypotheses were tested in three protocols using brush samples of airway epithelium obtained from allergic asthmatics and control subjects.

## Methods

### Subjects and bronchoscopic protocols

Thirty-one individuals characterized as mild allergic (to ragweed) asthmatics based on American Thoracic Society criteria, and 17 nonasthmatic, normal healthy subjects volunteered for the studies approved by the Wake Forest University School of Medicine or the Jefferson Medical College Institutional Review Boards. All volunteers gave informed, written consent to screening, bronchoscopic procedures and characterization as previously described [13,14]. Asthmatics had mild intermittent asthma and 29 of the 31 were using only inhaled β_2_-agonist on an as needed basis; the remaining 2 asthmatics were using chronic inhaled steroid and long-acting β_2_-agonist therapy. These latter two subjects contributed bronchial epithelial cells only to "c" protocol (see below) and DNA for β-adrenergic receptor haplotyping. Characterization of all subjects included collection of demographic information, standard spirometry, airway reactivity to methacholine, skin reactivity to ragweed allergen (short ragweed antigen, Greer Laboratories, Lenoir, NC) as previously described [14], and β-adrenergic receptor haplotyping. Results for other investigations on some of the subjects in these studies have been reported: leukocytes and inflammatory mediators (TGFβ1, TGFβ2, IL-4, IL-10, IL-13, sphingosine 1-phosphate, sVCAM) in lavage fluid induced by segmental allergen challenge, TRAIL and its receptors, induction of stress proteins in epithelial cells, epithelial cell proliferation and stimulation of fibrogenesis, and β_2_-adrenergic receptor signaling [13-22]. Subject demographic and pulmonary function data are listed in Table 1.

**Table 1:** Subject demographics. Data are presented as mean ± SEM except for PC_20 _methacholine which are presented as the geometric means.

**Characteristics**	**Allergic Asthmatic**	**Nonasthmatic Normal**	**P Value**
**Number:**	31	17	
**Gender:**	17 M/14 F	12 M/5 F	
**Age **(yr): (range)	29.6 ± 1.3 (20 – 47)	27.1 ± 1.3 (21 – 33)	P = 0.22
**Ragweed Skin Tests**	Positive	Negative	
**FEV_1 _**(L):	3.33 ± 0.2	4.06 ± 0.2	P = 0.005
**FEV_1 _**(% predicted):	84.8 ± 6	101.3 ± 3.7	P = 0.07
**PC_20 _Methacholine **(mg/ml):	1.0 ± 0.8	16 at > 32 mg/ml 1 at 11 mg/ml	
**Serum IgE **(IU/ml):	177 ± 1	51 ± 13	P = 0.002

Bronchoalveolar lavage (BAL) and brush biopsies of bronchial epithelium from asthmatic or control subjects were obtained in three separate protocols: (***a***) before (designated as day 1) and 24 hr, 1 week and 2 weeks after segmental allergen challenge (SAC) (days 2, 9 and 16, respectively) in a recovery from injury protocol (Table 2) (n = 16: 9 asthmatics, 7 nonasthmatic normals); (***b***) before (day 1) and 24 hr after SAC (day 2) at baseline, repeated sampling before and 24 hr after SAC (days 18 and 19) after a two week period of regular inhaled β_2_-agonist (albuterol) or placebo, and final repeat sampling (day 25) of initial lung segments after 1 additional week of β_2_-agonist or placebo, withdrawn 12 hours prior to bronchoscopy (Table 3) (n = 7 asthmatics on β_2_-agonist and a repeat of n = 4 asthmatics on placebo); and (***c***) baseline bronchoscopy on asthmatics (n = 16) and nonasthmatic normals (n = 10). All subjects in protocols "**a**" and "**c**" received albuterol just prior to bronchoscopy; those subjects enrolled in "b" protocol received albuterol just prior to bronchoscopy only after the two-week period of regular inhaled β_2_-agonist (days 18 and 19). Bronchoscopy with allergen challenge (Ag challenge) was preformed as previously described in detail using 10 ml of a 100X concentration of the minimum antigen dose giving a positive skin test (wheal ≥ 10 mm with 0.001 RWAU/ml) [23]. For bronchoalveolar lavage, 3 aliquots of 50 mls sterile normal saline (warmed to 37°C) were instilled and removed by gentle suction. Brush biopsies of epithelium were obtained from 2–3 cytology brushings combined and processed as reported in detail and below [18,19].

**Table 2:** Recovery from injury bronchoscopy protocol. Samples (lavage and brush biopsies) from four bronchoscopic procedures on each of 9 asthmatic and 7 nonasthmatic normal subjects were obtained from unchallenged and antigen (Ag) challenged lung segments according to the procedure day as indicated. A second Ag challenge on day 2 was performed to provide an additional challenged segment for day 9 without compromise from prior sampling.

**Procedure Day:**		**1**	**2**	**9**	**16**
**Lung Segment:**					
Left Lower	(Unchallenged Seg)	Sample			
Right Middle	(Challenged Seg 1)	Ag Challenge	Sample		Sample
Lingula	(Challenged Seg 2)		Ag Challenge	Sample	

**Table 3:** β_2_-agonist or placebo regular inhalation and bronchoscopy protocol. Samples (lavage and brush biopsies) from five bronchoscopic procedures were obtained from the unchallenged and Ag challenged lung segments according to the procedure day indicated. Seven asthmatics enrolled in the drug arm, and 4 of these same subjects re-enrolled in the placebo arm. A baseline sample and a 24 hr post Ag challenge sample were obtained before (days 1 and 2) and after (days 18 and 19) a two week period of regular albuterol or placebo use. The initial baseline unchallenged and challenged lung segments were resampled following one additional week of regular albuterol or placebo use which were discontinued 12 hr before the final bronchoscopic procedure on day 25.

**Procedure Day:**		**1**	**2**	**3–17**	**18**	**19**	**20–24**	**25**
**Lung Segment:**								
**Lingula**	(Unchallenged)	Sample						Sample
**RML**	(Challenged)	Challenge	Sample					Sample
				Albuterol 2 puffs, qid				
**RLL**	(Unchallenged)				Sample			
**LLL**	(Challenged)				Challenge	Sample		
							Albuterol 2 puffs, qid	

Cells from BAL fluid were pelleted, resuspended and counted [21]. Aliquots of BAL cells (10^5 ^cells) from the autologous lung segment were added to medium in wells surrounding semiporous membrane culture inserts (0.4 μm pore size, Millicell-12 mm HA MCE; Millipore, Bedford, MA) containing the epithelial cell aliquots for co-culture. Cytospin preparations of BAL cells were stained, and differential counts for epithelial cells (Alcian Blue, ciliated, or other columnar cells, and their sum = "total" epithelial cell counts) were calculated (×10^4^/ml).

### Epithelial cell culture and western blot analysis of VASP

**Figure 1:**
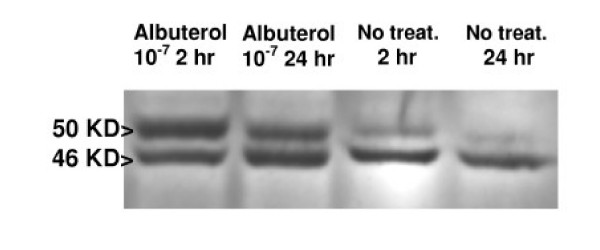
**Western blot of bronchial epithelial cells demonstrating 50 and 46 KD isoforms of VASP**. Representative western blot of bronchial epithelial cells from an asthmatic subject developed with anti-VASP antibody to show the banding pattern for the 46 KD and phosphorylated 50 KD isoforms of VASP. Bronchial epithelial cells received either no treatment for 2 or 24 hr or treatment with 10^-7 ^albuterol for 2 or 24 hr. The treatment with albuterol increased the band density for the 50 KD VASP isoform relative to the 46 KD isoform, greater at 2 hr than after 24 hr chronic albuterol treatment.

Bronchial epithelial cells were cultured 24 hr at air/fluid interface in Millicell culture inserts either without or with 10^5 ^BAL cells added to the well surrounding the insert to maintain contact with soluble inflammatory mediators from leukocytes of the same lung segment [18]. Inserts containing epithelial cells were harvested in RIPA buffer (phosphate buffered saline containing 1% NP40, 0.5% sodium deoxycholate, 1% sodium dodecyl sulfate, with protease inhibitors: 0.1 mg/ml PMSF, aprotinin and 1 mM sodium orthovanadate). Cell lysates were frozen until analyzed by SDS-PAGE and Western blotting [18,19]. Western blots were developed with monoclonal anti-VASP antibody, recognizing both the 46 kD and 50 kD (phosphorylated at Ser 157) molecular forms (Transduction Laboratories, Lexington, KY) (see Figure 1), and quantitated by densitometry (Kodak 1D Image Analysis System). This previously reported method [18] provides a linear response for band densities of the 50/46 KD VASP over the range of epithelial cell protein concentration examined.

To ascertain that epithelial cells of asthmatics were responsive to β_2_-agonists, aliquots of epithelial cells from some asthmatics were stimulated for 2 hr after the initial 24 hr incubation with *in vitro *addition of albuterol (10^-7 ^M), salmeterol (10^-7 ^M) or no stimulus before harvest and handling as described above.

### Haplotyping of β_2_-adrenergic receptor

DNA was isolated from cell pellets or lysates, amplified by GenomiPhi DNA kit (Amersham Biosciences, Piscataway, NJ)[24] if low in yield, and haplotyped for the β-adrenergic receptor according to the multiplex ARMS PCR assay [25]. DNA of 11 asthmatics and 7 nonasthmatic normals were sequenced by standard methodology [26] for the β-adrenergic receptor in addition to haplotyping to confirm haplotype assignment at the 13 SNP sites defined by Drysdale et al. [27].

### Statistical analysis

Data were tested for normal distribution and equal variances by Sigmastat (version 2.0) and examined by one- or two-way repeated measures analysis of variance, *post hoc *pairwise comparisons by Tukey test at individual points, paired or unpaired t test, as appropriate. A p < 0.05 was considered significant, and a 0.05 < p < 0.1, a trend.

## Results

### Allergen-induced inflammation effect on VASP phosphorylation

**Figure 2:**
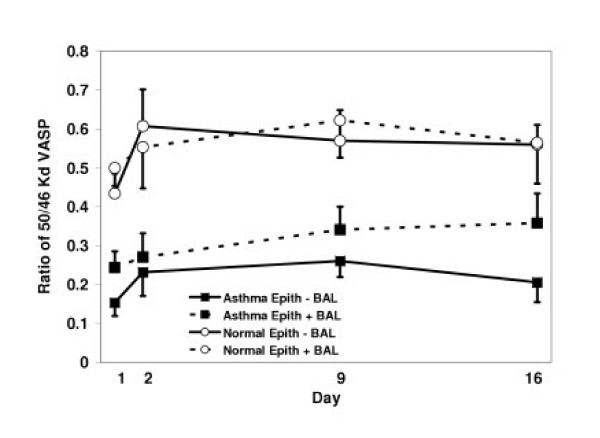
**VASP phosphorylation in epithelial cells of asthmatic and normal control subjects following allergen challenge**. Ratio of 50/46 KD VASP in epithelial cells of asthmatic (squares) and nonasthmatic normal subjects (circles) from before (Day 1) and after segmental allergen challenge (Days 2, 9 and 16). Epithelial cells were cultured for 24 hr with (dashed lines) or without (solid lines) BAL cell co-culture. Segmental allergen challenge increased the phosphorylation of VASP in asthmatic and normal epithelial cells but did not reach significance (p = 0.08). BAL cell co-culture exerted a significant effect on VASP phosphorylation in asthmatic (p = 0.022) but not normal epithelial cells. Nevertheless, asthmatic epithelial cells had significantly less VASP phosphorylation (lower 50/46 KD VASP ratio) at all timepoints compared to nonasthmatic normal epithelial cells (p < 0.001 without BAL cells; p = 0.006 with BAL cells).

To test the hypothesis that allergen-induced inflammation would increase phosphorylation of VASP, we examined the ratio of phosphorylated 50 KD to 46 KD VASP in epithelium from allergic asthmatic and nonallergic nonasthmatic normal subjects before and after segmental allergen challenge in the recovery from injury protocol (Table 2). The ratio of 50/46 KD VASP in epithelial cells of both asthmatic and normal subjects increased after Ag challenge (p = 0.08 for the effect of day on 50/46 KD VASP ratio), but did not reach significance in either group (Figure 2). Co-culture of epithelial cells with autologous BAL cells within the asthmatic subjects did significantly elevate the 50/46 KD VASP ratio (p = 0.022), but not within nonasthmatic normal subjects. The total VASP expression (46 KD + 50 KD relative to an internal 76 KD protein [18]) was also examined, but did not significantly differ between asthmatics' and normals' epithelial cells (p = 0.55), over the 4 timepoints (p = 0.87), or with BAL cell co-culture (p = 0.97) (data not shown). Asthmatic epithelial cells however, had a significantly lower ratio of 50/46 kD VASP compared to normal epithelial cells at baseline and throughout the two weeks post-challenge, both without BAL cell co-culture (p < 0.001) and with BAL cell co-culture (p = 0.006).

### Regular inhaled β_2_-agonist effect on VASP phosphorylation

**Figure 3:**
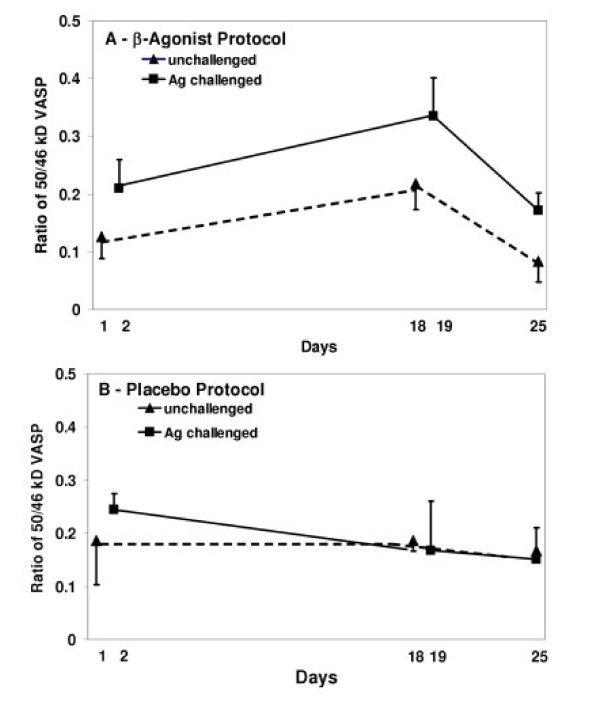
**VASP phosphorylation in epithelial cells of asthmatics after regular albuterol or placebo inhalation**. Ratio of 50/46 KD VASP in asthmatic epithelial cells without BAL cell co-culture from subjects either on regular albuterol inhalation (A) or on regular placebo inhalation (B). Epithelial cells were obtained from unchallenged lung segments (Days 1, and 18, broken lines), from Ag challenged lung segments (Days 2, and 19, solid lines) and from resampled lung segments on Day 25 (unchallenged Day 1 segment, and challenged Day 2 segment). Two weeks of regular β_2_-agonist inhalation, but not placebo inhalation, resulted in significantly increased phosphorylation of VASP in both unchallenged and challenged lung segments' epithelium (p = 0.009). Overall, VASP phosphorylation, i.e. a larger 50/46 KD VASP ratio, was greater in epithelium from challenged than from unchallenged lung segments in the regular β_2_-agonist inhalation protocol (p = 0.017), but not in the placebo inhalation protocol (B panel).

To test the hypothesis that regular inhalation of β_2_-agonist in asthmatics would increase VASP phosphorylation, we examined the ratio of 50 KD to 46 KD VASP before and after two weeks regular *in vivo *use of albuterol or placebo, and after an additional one week of regular use terminating 12 hr before the final bronchoscopy (Table 3). After two weeks inhaled regular β_2_-agonist use, the 50/46 KD VASP ratio was increased at day 18–19 for both unchallenged and Ag challenged segments' epithelium (effect for day, p = 0.009; day 18–19 versus day 1–2, p = 0.07; day 18–19 versus day 25, p = 0.008), but not after placebo (p = 0.79) in epithelial cells without BAL cell co-culture (Figure 3). The VASP ratio overall was greater in the Ag challenged lung segment versus the unchallenged lung segment (p = 0.017) in the β-agonist protocol (Figure 3A), but there was no statistical interaction for the effect of day with the lung segment, either challenged or unchallenged.

To confirm that the epithelial cells from asthmatics responded to both short and long-acting β_2_-agonists, aliquots of epithelium from four asthmatics at baseline were stimulated *in vitro *with either 10^-7 ^M albuterol, 10^-7 ^M salmeterol or unstimulated for 2 hr, and analyzed for 50/46 KD VASP. The VASP ratio in epithelial cells of all asthmatics was significantly increased by albuterol compared to unstimulated, but not by salmeterol (50 KD/46 KD VASP: 10^-7 ^M albuterol = 0.267 ± 0.06; 10^-7 ^M salmeterol = 0.36 ± 0.14, unstimulated = 0.11 ± 0.04; p = 0.018 for albuterol vs unstimulated, p = 0.13 for salmeterol vs unstimulated).

**Figure 4:**
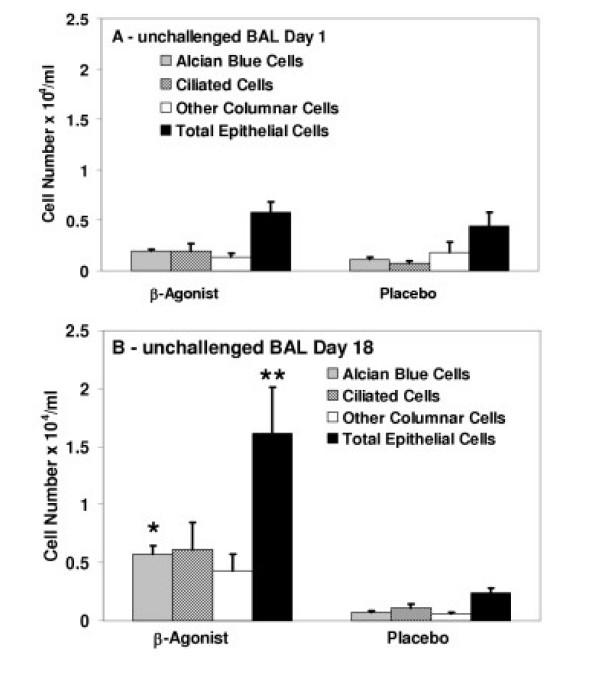
**Numbers of epithelial cells in control segment BAL fluid from asthmatics before and after regular β-agonist or placebo inhalation**. Epithelial cell numbers in unchallenged lung segment BAL fluid prior to any inhalation (A, day 1) and in unchallenged lung segment BAL fluid after two weeks regular inhalation of albuterol or placebo (B, day 18). The numbers of cells stained with Alcian Blue for mucous (gray shaded bars), nonspecific esterase for ciliated (hatched bars), other columnar cells (open bars) and the total of these three (solid bars) per ml were increased after two weeks of regularly inhaled albuterol compared to placebo (p < 0.001 for both Alcian blue and total groups). There was also a significant effect for day in Alcian blue stained cells (day 1 versus day 18, p = 0.014) and a significant group × day interaction in Alcian blue and total cell groups for albuterol inhalation (p = 0.004 and p = 0.014, respectively).

To determine whether the significant increase in VASP phosphorylation resulted in altered epithelial cell attachment, the numbers of epithelial cells in bronchoalveolar lavage fluid were quantitated and compared between regular albuterol use and placebo. Cells in lavage fluid from unchallenged lung segments were examined to avoid epithelial shedding due to the allergen-induced inflammation in challenged segments. Day 18 BAL leukocyte differential counts did not change from baseline, indicating no spillover of inflammatory response into the unchallenged lung segment (p = 0.44 for eosinophils, p = 0.56 for neutrophils, p = 0.28 for lymphocytes). Significantly increased numbers of Alcian blue-stained, mucin containing epithelial cells were observed in the lavage fluid of unchallenged segments of asthmatics after 2 weeks of β_2_-agonist, but not placebo (p < 0.001 for group, p = 0.014 for day and p = 0.004 for group × day interaction) (Figure 4). The number of mucin-containing cells correlated with the 50/46 KD VASP ratio observed (R = 0.577, p = 0.049). Concomitantly, significantly increased total epithelial cell numbers in lavage fluid were observed for β_2_-agonist but not placebo (p < 0.001 for group, and p = 0.014 for group × day interaction).

### β_2_-adrenergic receptor haplotype and VASP phosphorylation

Different haplotypes of the β_2_-adrenergic receptor are reported to alter receptor expression and *in vivo *responsiveness [27]. Thus, we determined β_2_-adrenergic receptor haplotypes of the asthmatic and nonasthmatic normal subjects, to examine whether the differences in baseline epithelial VASP phosphorylation between asthmatic and normal subjects might be due to increased representation of a particular β_2_-adrenergic receptor haplotype. Additional asthmatic and normal subjects, for whom both haplotype and 50/46 KD VASP data were available, were added to above subjects (Table 4). The overall 50/46 KD VASP ratio (mean ± sem) was significantly increased in nonasthmatic normal versus asthmatic subjects (0.38 ± 0.02 versus 0.16 ± 0.01, respectively; p < 0.001, two-tailed *t *test) as observed above (Figure 2). Asthmatic subjects tended to have several less frequent β_2_-adrenergic receptor haplotypes (for example: 1, 9, 10, and 12) than normals, and more often had the 4/4 homozygous haplotype, which has arginine at amino acid 16 [25,27]. The 4/4 haplotype is reported to be less responsive to β_2_-agonist [27]. Nevertheless, these haplotypes did not appear to substantially effect the 50/46 KD VASP ratio. A comparison of the more frequent 2/4 heterozygous haplotype for the 50/46 KD VASP ratio still remained different: 0.41 ± 0.07 nonasthmatic normals versus 0.16 ± 0.04 asthmatic subjects (p = 0.002, two-tailed *t *test).

**Table 4:** Mean of 50 KD/46 KD VASP ratio in epithelial cells according to β_2_-adrenergic receptor haplotype. Specific β_2_-adrenergic receptor haplotypes are shown with the mean ratio of 50/46 KD VASP for nonasthmatic normals or asthmatics having that haplotype. Asthmatics tended to have haplotype 4 which contains Arg at amino acid 16, and rarer haplotypes such as 1, 9, and 10 than nonasthmatic normals. The overall reduced phosphorylation of VASP (higher 50/46 KD ratio) in asthmatics compared to normals does not appear associated with a specific haplotype.

**Haplotype**	**2/2**	**2/4**	**2/6**	**4/4**	**4/6**	**6/10**	**1/1**	4/(1,9,12)*	**4/10**	**1/10**	**6/9**
***Normal*:**											
**Total N = 17**	N = 6	N = 5	N = 3	N = 1				N = 1	N = 1		
**Mean 50/46 KD**	**.307**	**.408**	**.455**	**.475**				**.168**	**.491**		
**± SEM**	**± 0.07**	**± 0.07**	**± 0.10**								
***AsthmaTic*:**											
**Total N = 30**	N = 2	N = 9		N = 8	N = 3	N = 1	N = 2	N = 3		N = 1	N = 1
**Mean 50/46 KD**	**.230**	**.163†**		**.164**	**.147**	**.187**	**.233**	**.110**		**.094**	**.046**
**± SEM**	**± 0.01**	**± 0.04**		**± 0.03**	**± 0.07**		**± 0.00**	**± 0.02**			

*The haplotyping method could not distinguish among these infrequent haplotypes (1, 9, and 12)

†Asthmatics versus Normals, p = 0.002 (two-tailed *t *test, equal variance)

## Discussion

Recent investigations into asthma pathogenesis have begun to focus, not on the acute inflammatory events involved with asthmatic airway inflammation, but on aberrant repair mechanisms which appear to be present [28-30]. This work focused on vasodilator-stimulated phosphoprotein (VASP) which is predicted to be involved in epithelial repair mechanisms by mediating focal adhesion, actin filament binding and polymerization, and ultimately, epithelial cell mobility. Reports published during the course of our studies have shown that detachment of kidney epithelial cells increases protein kinase A activity and its phosphorylation of VASP [12], as well as a transient increase in VASP expression [31]. Although there was some increase in total VASP (46 KD + 50 KD forms) in asthmatics compared to normals on day 2 in the recovery from injury protocol, the difference was not significant, and probably does not contribute substantially to cell adherence and motility. We hypothesized that asthmatics would demonstrate increased VASP phosphorylation compared to nonasthmatic, normal subjects to permit actin remodeling and cell migration for repair of inflammatory injury. Instead, we observed a potential defect as shown by decreased VASP phosphorylation in asthmatic epithelial cells prior to and following allergen-induced injury compared to normal subjects. Increased VASP phosphorylation in response to segmental antigen challenge was modest in both asthmatic and nonasthmatic normal epithelial cells, but overall a profound decrease in the VASP phosphorylation was observed in asthmatic epithelial cells at all time points examined. The reduced VASP phosphorylation in epithelial cells of asthmatics was confirmed by expanded examination of an additional group of subjects, both asthmatic and normal (Table 4). It has been previously shown that blocking of phosphorylation at MENA Ser 236, which corresponds in molecular structure to VASP Ser 157, or depletion of ENA/VASP results in increased cell spreading and reduced functional control of cell motility in a number of model systems [3-5,11]. More directly, 10 μM PGE_1 _converts 60% of the 46 KD VASP to 50 KD VASP and completely inhibits platelet aggregation as a measure of cell-cell adhesion [6]. Thus, even partial conversion of VASP to its phosphorylated form has significant impact. This suggests one potential mechanism for aberrant epithelial repair in asthmatics: defective or diminished VASP phosphorylation may indicate abnormal epithelial motility. Confirming defective epithelial cell motility in asthmatics *in vivo *will be challenging, but is a necessary next step in this work.

While segmental antigen challenge did not significantly increase VASP phosphorylation in the recovery from injury protocol "a", there was a trend toward a significant increase, which was confirmed in asthmatics enrolled in the regular β-agonist inhalation protocol "b". In addition, leukocytes in bronchoalveolar lavage fluid from asthmatics co-cultured with autologous epithelium did significantly increase VASP phosphorylation, unlike the leukocytes from nonasthmatic normal subjects, suggesting a soluble signal from the BAL leukocytes to epithelial cells in asthmatics. What the signal(s) may be remains to be determined, but other work from our laboratory suggests certain growth factors such as TGFβ, could be involved.

Because VASP becomes phosphorylated by cAMP-dependent protein kinase, we also hypothesized that both *in vivo *and *in vitro *exposure of epithelial cells to a β-agonist, which increases cyclic AMP levels, should increase VASP phosphorylation. As predicted, regularly inhaled albuterol increased VASP phosphorylation *in vivo *and apparently altered epithelial cell adhesion, producing significantly greater numbers of epithelial cells shed into bronchoalveolar lavage fluid from unchallenged lung segments exposed only to the β-agonist, without any allergen-induced inflammation. The β-agonist effect on VASP phosphorylation was short-lived and the ratio of 50/46 KD VASP returned to baseline within 12 hr of the last albuterol inhalation in vivo. Whether the increased epithelial cell detachment induced by albuterol inhalation returns to baseline as quickly has not been determined. β-agonist use may therefore produce conflicting effects on asthmatic epithelium. β-agonists inhibit keratinocyte migration by β_2_-adrenergic receptor activation of the serine/threonine phosphatase PP2A [32], the principal phosphatase which dephosphorylates VASP [33], and at the same time activate cAMP-dependent protein kinase phosphorylation of VASP [6-9]. Thus, β-agonists may promote epithelial repair by enhancing both phosphorylation and dephosphorylation of VASP in actin filament restructuring, but may also promote epithelial damage by increasing the detachment of epithelial cells from the airway. These results also caution against considering as "baseline" or "control," samples obtained from asthmatics receiving β-agonist therapy without an appropriate washout interval.

Genetic variation and altered function of the β2-adrenergic receptor could potentially contribute to the differences in VASP phosphorylation observed between asthmatics and nonasthmatic normal subjects [27]. However, examination of 50/46 KD VASP ratio grouped according to β2-adrenergic receptor haplotype for our study subjects did not reveal any specific haplotype bias, either homozygous or heterozygous, affecting VASP phosphorylation. Although the numbers of subjects in any one haplotype classification were limited, the largest 2/4 haplotype group, nonasthmatic normal subjects showed a significantly increased ratio compared to asthmatic subjects. It is possible that other characteristics of the β2-adrenergic receptor gene (for example, stability of message due to variation in the 3' untranslated region, [34]), variation in other components in the signaling cascade such as cAMP dependent protein kinase A [6-9], or activation of protein kinase C [10], genetic variation in VASP itself, or differences in the activity of protein phosphatases [33] between asthmatics and normals may contribute to the observed disparity in VASP phosphorylation here between asthmatic and normal subjects. Work is currently addressing these areas of inquiry.

## Conclusion

Phosphorylation of VASP is significantly reduced in bronchial epithelial cells from asthmatics compared to nonasthmatic normal subjects, although inducible by β-agonist treatment either in vitro or in vivo. The reduced ratio of phosphorylated to unphosphorylated VASP in asthmatics does not appear associated with genetic variation in the β_2_-adrenergic receptor. Regular inhalation of β-agonist results in increased VASP phosphorylation in epithelial cells and increased epithelial cell detachment from the airways.

## List of abbreviations

VASP, vasodilator-stimulated phosphoprotein; ENA, ENAbled; MENA, mammalian ENAbled; EVL, ENA/VASP-like; BAL, bronchoalveolar lavage; SAC, segmental allergen challenge;

## Competing interests

The author(s) declare that they have no competing interests.

## Authors' contributions

ATH conceived the study, supervised data collection, statistical analysis and interpretation, and drafted the manuscript. MW and KAR processed samples from bronchoscopy, and analyzed VASP data from western blots. GCF processed DNA from subjects, determined β-adrenergic receptor haplotype, and with GAH, sequence of β-adrenergic receptor; both provided drafting and critical comment on revision of the manuscript. RC, VB and JGZ consented and enrolled subjects, performed bronchoscopies, processed samples and critically commented on the manuscript. SPP participated in study design and coordination, analysis, writing and critical revision of the manuscript.
